# Another Opinion

**Published:** 1888-03-24

**Authors:** 


### ANOTHER OPINION.

The opinions of your readers having been asked in your last issue with regard to the proposed "British Nurses Association." I venture to write to say for myself, as a nurse here, and a number of my fellow-nurses, that we fully appreciate the scheme described in your article, and we heartily welcome the endeavour to form an association, being willing to become members whenever the opportunity occurs. I am sure the want is felt by all. Your article is very helpful, describing shortly the scheme, for nurses are as a rule always so engrossed with their work that they have not the time to attend meetings where they could hear about such things, and in the time they have off duty it is quite impossible to do much more than rest and attend to their needs; and in a large institution they seldom meet together except at the dinner hour, to talk over what might be for their future good. So any article putting things simply and shortly is invaluable to us. A Constant Reader.

[We shall be glad to have further letters on this subject. Will no one give the reasons in favour of the scheme of this Association?—Ed. *T. H.*]

